# Intraperitoneal administration of AAV9-shRNA inhibits target gene expression in the dorsal root ganglia of neonatal mice

**DOI:** 10.1186/1744-8069-9-36

**Published:** 2013-07-18

**Authors:** Akira Machida, Hiroya Kuwahara, Azat Mayra, Takayuki Kubodera, Takashi Hirai, Fumiko Sunaga, Mio Tajiri, Yukihiko Hirai, Takashi Shimada, Hidehiro Mizusawa, Takanori Yokota

**Affiliations:** 1 Department of Neurology and Neurological Science, Graduate School, Tokyo Medical and Dental University, Tokyo 113-8519, Japan; 2 Department of Orthopedic Surgery, Graduate School, Tokyo Medical and Dental University, Tokyo 113-8519, Japan; 3 Department of Biochemistry and Molecular Biology, Nippon Medical School, Tokyo 113-8602, Japan; 4 Core Research for Evolutional Science and Technology (CREST), Japan Science and Technology Agency (JST), Tokyo, Japan; 5 Department of Neurology and Neurological Science, Tokyo Medical and Dental University, 1-5-45 Yushima, Bunkyo-ku, Tokyo 113-8519, Japan

**Keywords:** RNA interference, Adeno-associated virus 9, Dorsal root ganglia, Blood–nerve barrier

## Abstract

**Background:**

There is considerable interest in inducing RNA interference (RNAi) in neurons to study gene function and identify new targets for disease intervention. Although short interfering RNAs (siRNAs) have been used to silence genes in neurons, *in vivo* delivery of RNAi remains a major challenge, especially by systemic administration. We have developed a highly efficient method for *in vivo* gene silencing in dorsal root ganglia (DRG) by using short hairpin RNA–expressing single-stranded adeno-associated virus 9 (ssAAV9-shRNA).

**Results:**

Intraperitoneal administration of ssAAV9-shRNA to neonatal mice resulted in highly effective and specific silencing of a target gene in DRG. We observed an approximately 80% reduction in target mRNA in the DRG, and 74.7% suppression of the protein was confirmed by Western blot analysis. There were no major side effects, and the suppression effect lasted for more than three months after the injection of ssAAV9-shRNA.

**Conclusions:**

Although we previously showed substantial inhibition of target gene expression in DRG via intrathecal ssAAV9-shRNA administration, here we succeeded in inhibiting target gene expression in DRG neurons via intraperitoneal injection of ssAAV9-shRNA. AAV9-mediated delivery of shRNA will pave the way for creating animal models for investigating the molecular biology of the mechanisms of pain and sensory ganglionopathies.

## Background

Dorsal root ganglia (DRG) are nodules that contain the cell bodies of primary sensory neurons, which send out afferent axons and convey sensory information from the periphery to the spinal cord. The loss of sensory neurons in DRG causes the degeneration of peripheral axons and central sensory projections in the posterior columns. Dysfunction of small DRG neurons may cause intractable sensory symptoms, including severe pain and allodynia, and that of large neurons may cause motor symptoms due to sensory ataxia.

To investigate the mechanisms of pain and systemic sensory neuronopathies, it is necessary to establish methods other than knockout mouse techniques to postnatally regulate genes in the DRG, because genetic deletion of endogenous DRG genes may cause compensatory modulation of other genes during developmental or lead to embryonic lethality in mice [1].

RNA interference (RNAi) has emerged as a powerful tool to induce loss-of-function phenotypes through the post-transcriptional silencing of gene expression [2,3], not only because of its potential application in functional genomic studies and target validation, but also because of its potential use as a therapeutic strategy to silence disease-causing genes. The RNAi pathway is initiated by the Dicer enzyme, which cleaves long, double-stranded RNAs into short (21- to 23-nucleotide) interfering RNAs (siRNAs) that mediate sequence-specific gene silencing [4,5]. siRNAs can also be derived from short hairpin RNAs (shRNA) expressed from a viral vector [6].

Adeno-associated virus (AAV) vectors are an effective tool for delivering genes into the central nervous system (CNS) because of their ability to infect post-mitotic neurons and mediate efficient and stable transduction with little immunogenicity or toxicity [7,8]. A number of serotypes have been identified, which have varying efficiency and tropism [9,10]. Ourselves and another group have reported that, when administered intrathecally, AAV5 and AAV9 are efficient vehicles to deliver siRNA to DRG neurons [11,12]. Zheng et al. demonstrated that AAV8 is effective in retrograde transport from muscle to spinal cord and DRG [13]. Foust et al. reported that a single intravenous injection of self-complementary AAV9 into newborn mice provided gene transfer across the blood–brain barrier (BBB) and into the CNS [14]. Subsequently, other researchers have reported that intravenous administration of single-stranded AAV9 vector can deliver a target gene with extensive CNS expression [15,16]. However, all of these past reports are related to the expression of a reporter or functional protein; there have been no reports demonstrating silencing of a target gene in the nervous system via intraperitoneal injection.

In the present study, we injected single-stranded AAV9-shRNA (ssAAV9-shRNA) intraperitoneally to neonatal mice and sufficiently inhibited a target gene in the DRG without any side effects. We chose superoxide dismutase 1 (*SOD1*) as the target gene because it is an appropriate endogenous gene for evaluating the side effects associated with both shRNA and AAV administration. *SOD1* is ubiquitously expressed in the CNS, and *SOD1*-knockout mice exhibit no obvious neurological phenotypes [17,18].

## Results

### Efficient transfer of GFP to DRG after intraperitoneal administration of ssAAV9 vector

**Figure 1:**
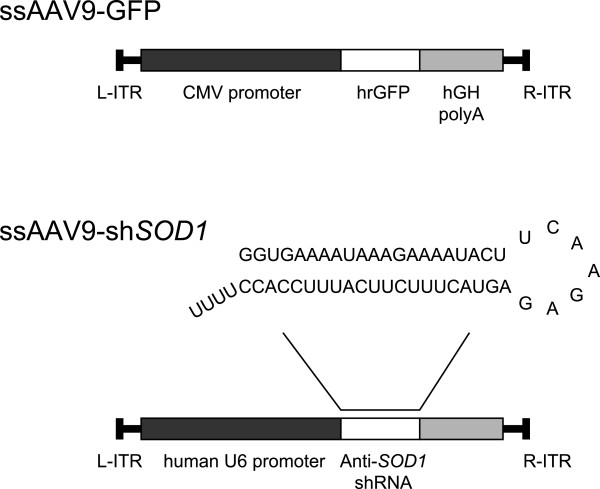
**Constructions of ssAAV9-GFP and ssAAV9-sh*SOD1 *vector.** CMV = cytomegalovirus, hrGFP = humanized Renilla Green Fluorescent Protein, hGH poly A = human growth hormone polyadenylation, L-ITR and R ITR = left and right inverted terminal repeats, respectively.

**Figure 2:**
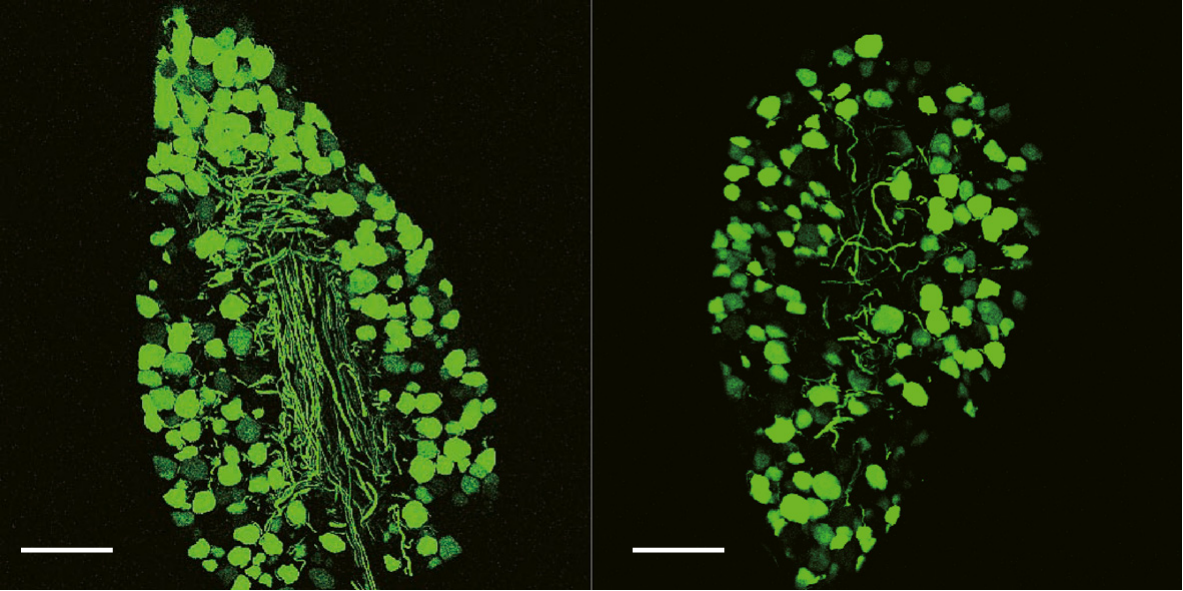
**Efficient gene transfer to DRG after neonatal intraperitoneal delivery.** Identification of efficient GFP expression in DRG 4 weeks after intraperitoneal injection of ssAAV9-GFP vector. Scale bars: 100 μm.

To investigate whether AAV9 vector can transfer a gene to DRG, we intraperitoneally injected neonatal mice with ssAAV9 vector encoding green fluorescent protein (ssAAV9-GFP, Figure 1). Four weeks after the injection, the mice were euthanized, and GFP expression in the DRG was assessed. We detected efficient GFP expression in the DRG (Figure 2), indicating that the ssAAV9 vector can deliver the GFP gene to the DRG. In the CNS, GFP expression was not detected except in the posterior column and posterior horn, into which the axons of the DRG neurons project (Additional file 1: Figure S1).

### *SOD1* mRNA and protein expression in DRG after intraperitoneal administration of anti-*SOD1* ssAAV9-shRNA (ssAAV9-sh*SOD1*)

**Figure 3:**
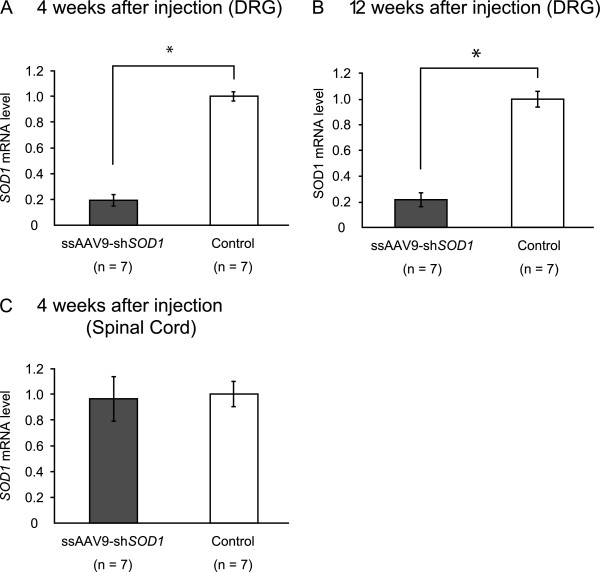
**Silencing effect of ssAAV9-sh*SOD1 *vector on mRNA expression level of *SOD1.*** The qRT-PCR of *SOD1* mRNA in DRG at 4 weeks **(A)** and 12 weeks **(B)** after injection with ssAAV9-shRNA vector shows that superoxide dismutase-1 (*SOD1*) expression was significantly inhibited (*P < 0.05). There is no significant difference of the expression level of *SOD1* mRNA between ssAAV9-sh*SOD1* and control in the spinal cord **(C)**.

**Figure 4:**
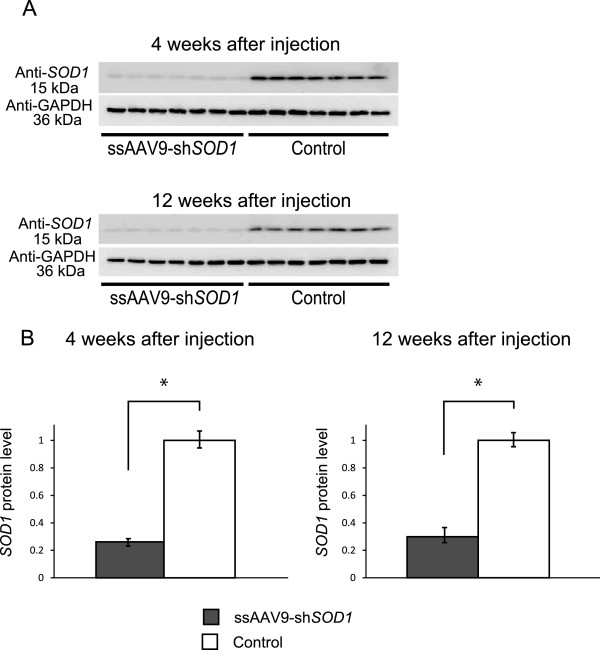
**Silencing effect of ssAAV9-sh*SOD1 *vector on protein level of *SOD1*.** The *SOD1* protein level was reduced in DRG as assessed by Western blot analysis at 4 weeks and 12 weeks after injection **(A)**. Densitometric analysis of Western blot bands confirmed significant downregulation of *SOD1* in the injected mice at both 4 and 12 weeks **(B)**. Data are presented as mean ± SEM (*P < 0.05, n = 7 for each group).

We next intraperitoneally injected neonatal mice with ssAAV9-sh*SOD1* vector to determine its ability to suppress the expression of *SOD1* in DRG (Figure 1). Four weeks after injection, *SOD1* expression in the DRG was assessed by quantitative RT-PCR (qRT-PCR). In order to evaluate the reduction level of *SOD1*, we used the DRG of ssAAV9-GFP injected mice as a control. The expression level of *SOD1* mRNA in the DRG of injected mice was about 80% lower than that of controls (Figure 3A) and lasted for at least 12 weeks (Figure 3B). We did not detect any *SOD1* silencing in the spinal cord (Figure 3C). The knockdown effect was specific for the target gene (*SOD1*), given that the levels of other endogenous mRNAs—those encoding GAPDH, hypoxanthine-guanine phosphoribosyltransferase, and β-tubulin—did not change (data not shown), suggesting that ssAAV9-sh*SOD1* did not affect the expression of unrelated endogenous genes. Western blot analysis confirmed decreased *SOD1* levels in the DRG of the mice injected with ssAAV9-sh*SOD1* (Figure 4A). Compared with that in control mice, the mean level of *SOD1* expression in anti-*SOD1* mice was 74.7% and 69.6% lower after 4 and 12 weeks, respectively (Figure 4B).

### Histological evaluation of DRG

**Figure 5:**
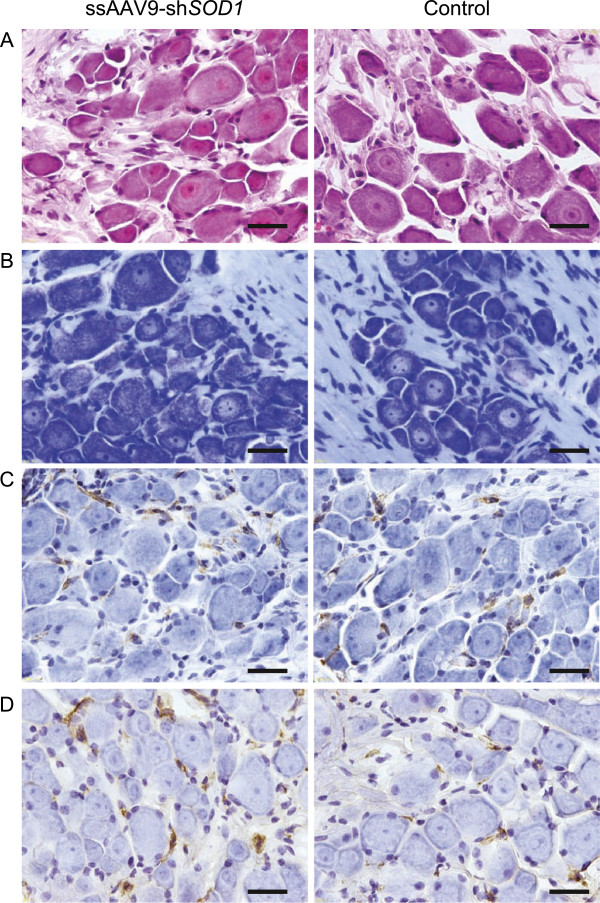
**Pathological examination of DRG.** Photomicrographs of hematoxylin and eosin staining **(A)**, Nissl staining **(B)**, Iba1 **(C)** and CD68 **(D)** immunohistochemical staining of DRG neurons from ssAAV9-sh*SOD1*–injected mice showed no differences between ssAAV9-sh*SOD1* and control (scale bars: 20 μm).

We histologically evaluated whether any anatomical abnormalities occurred in the DRG after ssAAV9-sh*SOD1* injection. Hematoxylin and eosin and Nissl staining of the DRG showed no inflammatory, necrotic, or degenerative lesions (Figure 5A, B). In order to confirm the neuroinflammation, we performed the following immunohistochemical staining; Iba1 (Figure 5C) for microglial activation and CD68 (Figure 5D) for macrophage invasion. Although we detected the slight microglial activation and macrophage invasion, there was no deference between AAV9-sh*SOD1* and control.

### Body weight and locomotive and sensory functions

**Figure 6:**
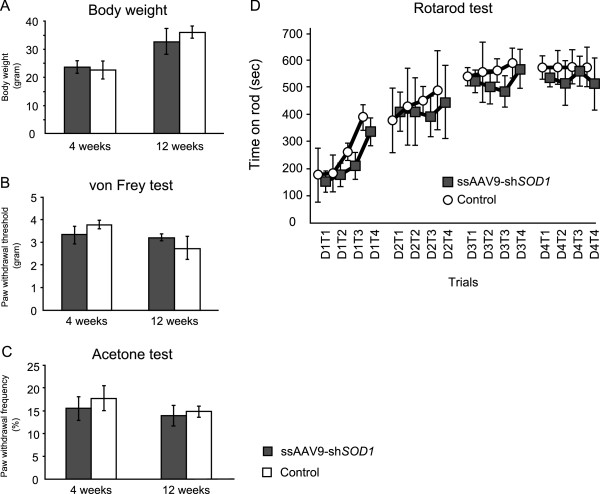
**Sensory and locomotive function and growth of the mice.** At 4 weeks and 12 weeks, none of the ssAAV9-sh*SOD1*–injected mice showed any significant differences in body weight compared with controls **(A)**. Similarly, in the von Frey test **(B)** and acetone test **(C)**, none of the injected mice showed significant differences in sensory function compared with controls. In the accelerating rotarod test, all of the injected mice could perform the task similar to that of the controls **(D)**. Each mouse was examined in 4 trials each day (T1–T4), for 4 consecutive days (D1–D4). There was no significant difference between the injected mice group and the control group. Data are presented as mean ± SEM (n = 7 for each group, *P* < 0.05).

To assess their general health and the motor and sensory functions of their hind limbs, we observed the mice at 4 and 12 weeks after injection with ssAAV9-sh*SOD1*. Body weights of the mice in the ssAAV9-sh*SOD1*–injected group were similar to those in the control group (Figure 6A). Importantly, there were no significant differences between the groups at 4 and 12 weeks after injection, according to tactile (Figure 6B) and acetone (Figure 6C) tests. Furthermore, accelerating rotarod tests revealed no significant differences in motor function between controls and ssAAV9-sh*SOD1*–injected mice (Figure 6D). We previously reported that the serum aspartate aminotransferase, alanine aminotransferase, alkaline phosphatase, lactate dehydrogenase, creatine kinase, total protein, and albumin values are similar in both groups [19]. All of these data demonstrate that the injected mice grow and develop without any side effects from the ssAAV9-sh*SOD1* vector.

## Discussion

We report that a single intraperitoneal injection of ssAAV9-sh*SOD1* inhibits *SOD1* expression in neonatal DRG without any adverse effects. Although the successful delivery of reporter genes (e.g., *GFP* and *LacZ*) and functional genes encoding therapeutic or pathogenic proteins (e.g., Survival of Motor Neuron, prepro-β-endorphin a, TAR DNA-binding protein 43) have been reported previously [13-16,20,21], our current data are the first to show sufficient inhibition of a target gene in neurons after a single intraperitoneal administration of vector-mediated delivery of shRNA. In previous reports, gene knockdown in DRG was achieved by direct injection into these structures [22] or by retrograde transport after sciatic nerve injection [23]. Recently, we and another group showed efficient gene knockdown in DRG after intrathecal delivery [11,12]. These approaches enable viral vectors to bypass the blood–nerve barrier (BNB) and are therefore suitable for silencing the DRG neurons local to where the vectors are injected. However, to utilize gene delivery methods in pain research, frequent and multiple injections are needed to transduce DRG neurons throughout the whole body, practice that leads to increased viral load and risk of an immune response [24]. In contrast, intraperitoneal delivery methods provide extensive and global inhibition of the target gene in all DRG neurons through a simple procedure. In this respect, the simplicity of intraperitoneal injection makes the technique superior for inducing RNAi in DRG neurons throughout the whole body.

In this study, we inhibited target gene expression in neonatal DRG neurons despite the known hepatotropism of AAV vectors after intraperitoneal administration. Although it is ultimately desirable to minimize target gene expression in adult mice, administration of high doses of AAV vectors interferes with endogenous microRNA processing, causing microRNA saturation and ultimately fatal liver toxicity because of the hepatotropism of AAV[25]. In the case of neonates, however, expression of shRNA in the liver is followed by rapid dilution of the shRNA during liver growth and hepatocyte division [26,27]. We previously demonstrated low reduction of target gene expression in neonatal liver by intraperitoneal injection of ssAAV9-shRNA [19], a finding that indicates that shRNA-induced silencing has different features in dividing compared with non-dividing cells and that neonatal DRG are a suitable target for intraperitoneally administered AAV9-shRNA.

Using intraperitoneal administration, we successfully bypassed the BNB and delivered ssAAV9-sh*SOD1* vector to DRG. The BNB is formed by the tight junctions between the endothelial cells of capillaries, and it restricts the transport and diffusion of solutes from blood to nerves [28-30]. We consider the following three possible routes for AAV9 to cross the BNB: (1) AAV9 directly crosses the BNB and reaches the DRG. Some evidence suggests that the barrier function of the BNB is especially vulnerable during the neonatal period, when the capillaries supplying the DRG are fenestrated, leading to a loose BNB that might not give full protection against toxins and antibodies [30]. Therefore, DRG exist outside of the protection of the BNB and are exposed to circulating solutes in the blood, including AAV vectors. (2) AAV9 enters the ventricles across the blood–cerebrospinal-fluid (CSF) barrier and is then distributed to DRG by the circulating CSF. Miyake et al. demonstrated that stronger GFP signals were detected in areas in contact with the CSF after intravenous injection of AAV9-GFP and suggested a promising mechanism by which AAV9 vectors pass through the BBB [15]. Furthermore, the perineurium of the DRG has fewer layers of perineurial cells and larger gaps than do peripheral nerves [31]. (3) AAV9 are transported from muscle to the DRG. Systemically delivered AAV9 may infect sensory axons innervating skeletal muscle and thus are transported to neurons within the DRG. Foust et al. suggested the possibility that transport from muscle to DRG occurs after systemic injection of AAV8 vector [32]. Although the precise mechanism by which AAV9 vectors pass through the BNB is unclear currently, AAV9 vectors have a specific and favorable character for the induction of RNAi in DRG.

With intraperitoneal administration of ssAAV9-shRNA, we could not detect any substantial silencing effect in the nervous system, except for in the DRG neurons. Considering that we inhibited gene expression only in DRG neurons, this method appears to have potential for pain research. The study of pain is an important area of biomedical research that is necessary for the development of treatments for intractable pain. Knockout animal models have produced remarkable advances in the understanding of the mechanisms of pain. Several molecules within DRG such as the δ-opioid receptor [33]; the transient receptor potential (TRP) ion channel family: TRPC, TRPV, TRPM, TRPA, TRPP, and TRPML [34]; and the NR1 [35] and NR2B [36] subunits of the N-methyl-D-aspartic acid receptor may work together in the development of pain-related symptoms. It has been suggested that the expression of some of these molecules (e.g., TRPV1 and TRPA1) largely overlaps various subsets of nociceptors and that these molecules are functionally linked to and interact with sensory neurons *in vivo*[34]. Therefore simultaneous silencing of multiple genes may be necessary to elucidate the mechanism of individual molecules in noxious sensation. However, it will lead to the embryonic lethal phenotype when multiple genes are silenced in knockout animal models. On the other hand, our method enables to target plural genes by injecting ssAAV9-shRNA vectors that silence different targets without any side effects. The advantage of our method of gene silencing is that it makes it possible to down-regulate multiple genes at once to investigate the compensatory interaction of these genes while escaping from the embryonic lethal phenotype of the genes.

Although we confirmed that the gene-silencing effect in DRG lasted for as long as 3 months in our mice, we did not examine the full duration of this silencing effect. Prior exposure to the AAV capsid reportedly leads to the activation of memory CD8^+^ T cells and results in the elimination of AAV capsid-harboring cells and the reduction of transgene expression [37]. However, these reports all used human subjects, which are naturally infected by AAV during childhood, and all experimental animals other than humans have shown long-term expression of the transgene after AAV-mediated gene transfer [37]. In addition, unlike in adults, there are no memory CD8^+^ T cells in the neonatal immune system. Furthermore, we previously demonstrated that shRNA-transgenic mice expressing the same shRNA used in the present study showed marked significant suppression of *SOD1* for more than 1 year [38]. Considering these combined results, we think that the ssAAV9-sh*SOD1* used in this report can stably achieve long-term suppression of target gene expression in DRG.

## Conclusions

We succeeded in silencing target gene expression in DRG after a single intraperitoneal injection of ssAAV9-sh*SOD1* vectors without any adverse effects, such as liver dysfunction, developmental abnormalities, and sensory disturbance. Intraperitoneal injection of AAV9 will be examined in further studies for potential uses in the development of treatments for diseases affecting DRG neurons. DRG transduction may have potential applications in models of chronic pain and other sensory neuronopathies.

## Methods

### Construction, production, and titration of the ssAAV9-sh*SOD1* vector

We prepared the anti-*SOD1* shRNA cassette as previously reported [39]. The anti-*SOD1* shRNA cassette was cloned downstream of polymerase III human U6 promoter in the AAV vector plasmid (Stratagene, La Jolla, CA, USA) (Figure 1). The silencing efficiency of the anti-*SOD1* shRNA sequence was verified by using several cultured cell lines and transgenic mice expressing the anti-*SOD1* shRNA, as previously described [38]. The human growth hormone polyadenylation (hGH poly A) cassette (Stratagene) was inserted downstream of the shRNA sequence for the titration assay using qRT PCR (Figure 1). The recombinant viral vector was produced according to the three-plasmid transfection protocol by using the calcium phosphate method as previously reported [40]. AAV vectors were purified using ammonium sulfate precipitation and iodixanol (Axis-Shield, Norton, MA) continuous gradient centrifugation. Genome titers of the AAV vectors were determined by qRT-PCR using the TaqMan system [41]. The following primers and probes targeting the poly(A) signal were used: 5’-CAGGCTGGTCTCCAACTCCTC-3’ and 5’-GCAGTGGTTCACGCCTGTAA-3’ served as the primer set, and 5’-TACCCACCTTGGCCTC-3’ served as the probe.

### Animals

All of the animal procedures were performed in accordance with the protocols approved by the Animal Experiment Committee of Tokyo Medical and Dental University (protocol nos., 0100101 and 0130113A). Imprinting control region (ICR) mice were obtained from Oriental Yeast Co. Ltd. (Tokyo, Japan). Postnatal day-1 ICR mice were injected intraperitoneally with a volume of 200 μl (1.0 × 10^13^ viral genomes/ml) of ssAAV9-sh*SOD1* vector (n = 7). ssAAV9-GFP injected mice were used as the control group (n = 7). The body weights of the mice were measured throughout the experimental period. At 4 or 12 weeks after injection, all of the mice were euthanized after performing rotarod tests and a series of sensory behavioral tests. DRG, spinal cord and brain were collected for analysis.

### Measurement of RNA reduction by qRT-PCR

Total RNA was extracted from the collected tissues by using Isogen (Nippon Gene, Toyama, Japan). DNase-treated total RNA (0.2 μg) was reverse-transcribed by using SuperScript III Reverse Transcriptase (Invitrogen, Carlsbad, CA, USA). Complementary DNA (cDNA) was amplified by means of the quantitative *Taq*Man system on a Light Cycle 480 Real-Time PCR Instrument (Roche) according to the manufacturer’s protocol. The *SOD1* mRNA expression level in each tissue was measured by using the following primers and probe: forward primer, 5′-GGTGCAGGGAACCATCCA-3′; reverse primer, 5′-CCCATGCTGGCCTTCAGT-3′; and probe, 5′-AGGCAAGCGGTGAACCAGTTGTGTTG-3′. To normalize the real-time PCR values, cDNA was also amplified quantitatively with the TaqMan primers and probe sets for glyceraldehyde 3-phosphate dehydrogenase (GAPDH; Applied Biosystems, Warrington, UK). The ratio of the *SOD1* mRNA expression level to the GAPDH expression level was calculated to estimate the shRNA silencing efficiency. Significant differences between the two groups were calculated with Welch’s *t*-test and significance was defined as *P* < 0.05.

### Western blotting

Proteins were extracted from DRG samples. The tissues were homogenized in cold homogenization buffer containing 0.1% sodium dodecylsulfate (SDS), 1% sodium deoxycholate, 1% Triton X-100, and 1 mM phenylmethylsulfonyl fluoride, together with a protein inhibitor cocktail (Roche). A 4-μg aliquot of the protein extracted from each sample was mixed with Laemmli sample buffer (BioRad, Hercules, CA, USA), denatured at 95°C for 5 min, and separated on a 15% SDS–PAGE gel. The separated proteins were transferred to a polyvinylidene difluoride membrane (BioRad) and incubated with rabbit anti-*SOD1* antibody (StressGen Biotechnologies, Victoria, British Columbia, Canada) and mouse anti-GAPDH monoclonal antibody (Biodesign, Saco, ME, USA) as the primary antibody. After incubation, the membrane was rinsed and incubated with 0.1% horseradish peroxidase (HRP)–conjugated secondary antibodies, goat anti-rabbit HRP IgG, and goat anti-mouse HRP IgG (Thermo Science, Rockford, IL, USA). Protein–antibody interactions were visualized by using Supersignal West Femto Maximum Sensitivity Substrate (Thermo Science). The quantification of the band intensity was measured by NIH Image J software (version 1.47). We calculated the relative expression levels and performed the statistical analysis.

### Pathological examinations

Mice were anesthetized and the perfused with PBS followed by 4% paraformaldehyde. The DRG and spinal cords were the dissected out, fixed again overnight at 4°C, and immersed in PBS containing 30% sucrose. Thereafter, the specimens were placed in OCT compound (Tissue-Tek, Sakura Finetek, Japan) and snap-frozen in liquid-nitrogen, and tissue sections were cut to a thickness of 10 μm with LEICA CM3050 S cryostat (Leica Microsystems, Wetzlar, Germany) and mounted on glass slides. DRG and spinal cord were stained with hematoxylin and eosin and Nissl staining. GFP was detected by means of native fluorescence using LSM 510 (Carl Zeiss MicroImaging GmbH, Göttingen, Germany). For immunostaining with Iba1 and CD68, blocking of endogenous peroxidase (1% H_2_O_2_ in PBS for 30 min), heat induced epitope retrieval was applied. Sections were heated in citrate buffer in a microwave oven at 100% power (700 W) for 9 min, then left to cool down at room temperature. After rinsing in ddH_2_O and PBS, sections were blocked in 10% Normal Goat Serum and incubated with polyclonal rabbit anti-Iba1 antibody (1:500, Proteintech) and monoclonal mouse anti-human CD68 antibody (Dako) overnight at 4°C. The sections were then rinsed in PBS and incubated with the horseradish peroxidase-labeled polymer (N-Histofine Simple Stain MAXPO, Nichirei) for 1 h at room temperature. After rinsing again, the immunohistochemical reactions was visualized using 3,3’-diaminobenzidine tetroxide (DAB-4HCl, Dojin Kagaku). The sections were then counterstained with hematoxylin.

### Rotarod test

The rotarod test was performed by using an accelerating rotarod (Ugo Basile Biological Research Apparatus, Varese, Italy). The 12-week-old mice in both groups were placed on the rod (diameter, 3 cm) in four trials each day, for a series of 4 days. Each trial lasted a maximum of 10 min; the time spent on the rod without falling was recorded. The average time for each group was calculated, and statistical significance was assessed by one-way ANOVA with repeated measures. Significance was defined as *P* < 0.05.

### Sensory behavioral tests

The animals were placed in Plexiglas boxes (9.5 × 21 × 25 cm) to become acclimated to the testing environment. These boxes were then placed on an elevated perforated plastic surface for at least 30 min before all behavioral tests [42]. A blinded observer conducted the behavioral testing.

### Tactile threshold

Mechanical sensitivity was measured by applying a series of calibrated von Frey filaments (0.02–8 g) to the plantar aspect of a hind paw. Each filament was applied once to each mouse. Beginning with the 1-g filament, each filament was applied perpendicularly to the hind paw for 4 to 6 sec. Brisk withdrawal of the hind paw indicated a positive response, and a lack of withdrawal indicated a negative response. This filament testing was repeated a maximum of two additional times, and at least two positive responses among the three applications of the filament indicated an overall positive response. If the mouse demonstrated an overall positive response, the next lower force filament was applied as described previously. If no overall positive response was observed (zero or one response in three), the next greater force filament was applied as described previously. Once the crossover threshold could be determined (i.e., from response to no response, or vice versa) the responses to the next five filaments were recorded to determine the median withdrawal threshold.

### Response to acetone

By using a plastic tube connected to a 1-ml syringe and without touching the skin, 100 μl of acetone was applied to the plantar surface of the paw. Acetone was applied five times to each paw at an interval of at least 30 sec, and the number of brisk foot withdrawals in response to the acetone application was recorded.

### Statistical analysis

All data are presented as means –standard error of mean (SEM; n =7). We performed D’Agositino-Pearson normality test and confirmed a normal distribution for performing parametric analyses. We performed the statistical analysis by Student t test for comparisons between the two groups for all experiments, with the exception of the Rotarod test. For the Rotarod test, the average time of each group was calculated, and statistical significance was assessed by one-way ANOVA with repeated measures. Significance was defined as p values less than 0.05.

## Abbreviations

RNAi: RNA interference; siRNAs: Short interfering RNAs; shRNA: Short hairpin RNAs; DRG: Dorsal root ganglia; AAV: Adeno-associated virus; SOD1: Superoxide dismutase 1; ssAAV9-shRNA: Single-stranded short hairpin RNA–expressing adeno-associated virus 9; CNS: Central nervous system; BNB: Blood–nerve barrier.

## Competing interests

The authors declare that they have no competing interests.

## Authors’ contributions

AKM designed and involved in all experimental procedures, performed statistical analyses, contributed to graphics preparation, and wrote the manuscript. HK initiated the studies and contributed to the experimental design and performed real-time PCR experiments. AZM initiated the studies. TK contributed to experiment design and supervised research. TH performed surgery to collect DRG samples. FS created and tested the ssAAV9-GFP and ssAAV9-shRNA vector. MT performed pathological examinations. YH and TS supervised the production of AAV vectors. HM commented on manuscript drafts. TY organized the team, contributed to the experimental design, and revised the manuscript. All authors have read and approved the final manuscript.

## Supplementary Material

Additional file 1: Figure S1 Gene transfer to spinal cord after neonatal intraperitoneal delivery. GFP expression was not detected except in the posterior column and posterior horn (A) and there was no GFP expression in the anterior side of the spinal cord (B). High magnification views of boxed area in (A) was shown in (C). Arrow indicates the posterior column and * indicates central canal. The nucleus of the spinal cord was stained with DAPI. Click here for file
